# Toxic effects of Arianor Ebony hair dye on human cells

**DOI:** 10.1590/1414-431X2023e12777

**Published:** 2023-07-21

**Authors:** Y. Tafurt-Cardona, P. Suares-Rocha, B.O. Silva, K.C.M. Moraes, M.A. Marin-Morales

**Affiliations:** 1 Departamento de Biologia Geral e Aplicada, Instituto de Biociências, Universidade Estadual Paulista, Rio Claro Campus, Rio Claro, SP, Brasil; 2 Facultad de Ciencias de la Salud, Fundación Universitaria Navarra - UNINAVARRA, Neiva-Huila, Colombia; 3 Centro Universitário FUNCESI, Itabira, MG, Brasil

**Keywords:** Temporary black hair dye, Comet assay, Micronucleus test, Gene expression

## Abstract

To evaluate the risks of hair dye exposure, we investigated cellular and molecular effects of Arianor Ebony dye, which is a mixture of azo and anthraquinone dyes, used in the composition of the black color. Cytotoxicity, genotoxicity, and gene expression of relevant molecules of apoptotic and oxidative stress mechanisms were investigated in HepG2 cells exposed to Arianor Ebony. Results showed that the dye did not induce cytotoxicity to exposed cells at a concentration up to 50 µg/mL compared to the negative control. However, genotoxic assays indicated that the dye was able to damage the genetic material at a concentration of 25 µg/mL, with induction factor values of exposed cells two- to five-fold higher than those recorded for the negative control. Moreover, the lowest observed effect concentration was 12.5 µg/mL. For gene expression, relevant changes were observed in *cytochrome c* and *caspase 9*, which decreased in cells incubated with the dye in a dose-dependent manner when compared with the negative control. In parallel, the expression of genes for antioxidant enzymes was increased in exposed cells, suggesting the presence of metabolic routes that protect cells against the toxic effect of the dye, avoiding exacerbated cellular death. Results suggested that the dye disrupted cellular homeostasis through mitochondrial dysfunction, which may be hazardous to human health. Thus, further investigations are necessary to deeply understand the mechanisms of action of the dye, considering its toxic potential found in our *ex vivo* assays.

## Introduction

Dyes are widely used in clothes, paper, cosmetics, pharmaceuticals, food, and even hair dyes. The hair dye industry contributes to the economic sector of our society and more and more people dye their hair (1). The scientific community is increasingly concerned about the use of these products, as they are composed of several chemicals, such as aromatic amines, which may be associated with the risk of developing cancer (2). According to *in vitro* and *in vivo* studies, aromatic amines are potentially genotoxic, mutagenic, and/or carcinogenic. When present in hair dyes, they can be absorbed through the skin during handling and application of the product and then be metabolized in the liver (3). The metabolism and reactivity of intermediate metabolites are the main factors that determine the genetic damage associated with aromatic amines (4). In addition, biochemical evidence indicates that carcinogenic substances present in dyes can damage exposed cells, which may undergo apoptosis (5).

Black hair dye is composed of a mixture of pure basic dyes. Arianor Ebony (Sensient Cosmetic Technologies, Brazil) is a mixture of Basic Blue 99, Basic Brown 16, Basic Red 76, Basic Yellow 57, and Acid Violet 43, which are commonly used in hair products. Basic Blue 99 contains 23 to 32 substances, and the Ames test demonstrated that it had mutagenic potential, inducing frameshift mutations (6). Basic Brown 16 presented clastogenic effects in mice and induced gene mutation in bacteria (7). Basic Red 76 induced micronuclei in an *in vitro* test in dermal cells (8). Acid Violet 43 presented potential clastogenic/cytotoxic effects in other analyses (9). These compounds are chemicals classified as azo dyes or anthraquinones, which may be toxic and deleterious to animals and humans (10). However, anthraquinones are widely used for many applications (staining of cellulose fibers, drugs, food, among others) and are the second largest class of textile dyes (15%), after azo dyes (70%) (10). Considering their use in hair dyes, public health regulatory agencies including the International Agency for Cancer Research (IARC) demonstrated that these cosmetics are considered mutagenic and carcinogenic (IARC, 1993) (11).

Due to the toxic characteristics of Arianor Ebony and its broad use, especially in black hair color, the present *in vitro* study investigated the cytotoxic and genotoxic effects of this dye in the hepatocarcinoma human cell line HepG2. Since it is metabolic-competent, HepG2 cells do not need the addition of exogenous triggers (12), being activated by some phase I and phase II enzymes related to the metabolism of chemical substances (13). Several researchers utilized HepG2 cells as test systems in the assessment of genotoxic potential of various test substances using the comet assay and micronucleus test (MN) (14,15). Acute cytotoxicity was assessed by the thiazolyl blue tetrazolium bromide (MTT) test, a colorimetric assay that evaluates the metabolic activity of living cells. The genotoxic potential was assessed by the comet assay, an electrophoretic technique that detects DNA strand breaks in individual cells, and MN test, which detects chromosome damage. In addition, expression levels of genes relevant for apoptosis and antioxidant mechanisms were measured to evaluate the effects of the dye in exposed HepG2 cells to assess the molecular pathways and physiological effects.

## Material and Methods

### Test substance - Arianor Ebony dye

**Figure 1:**
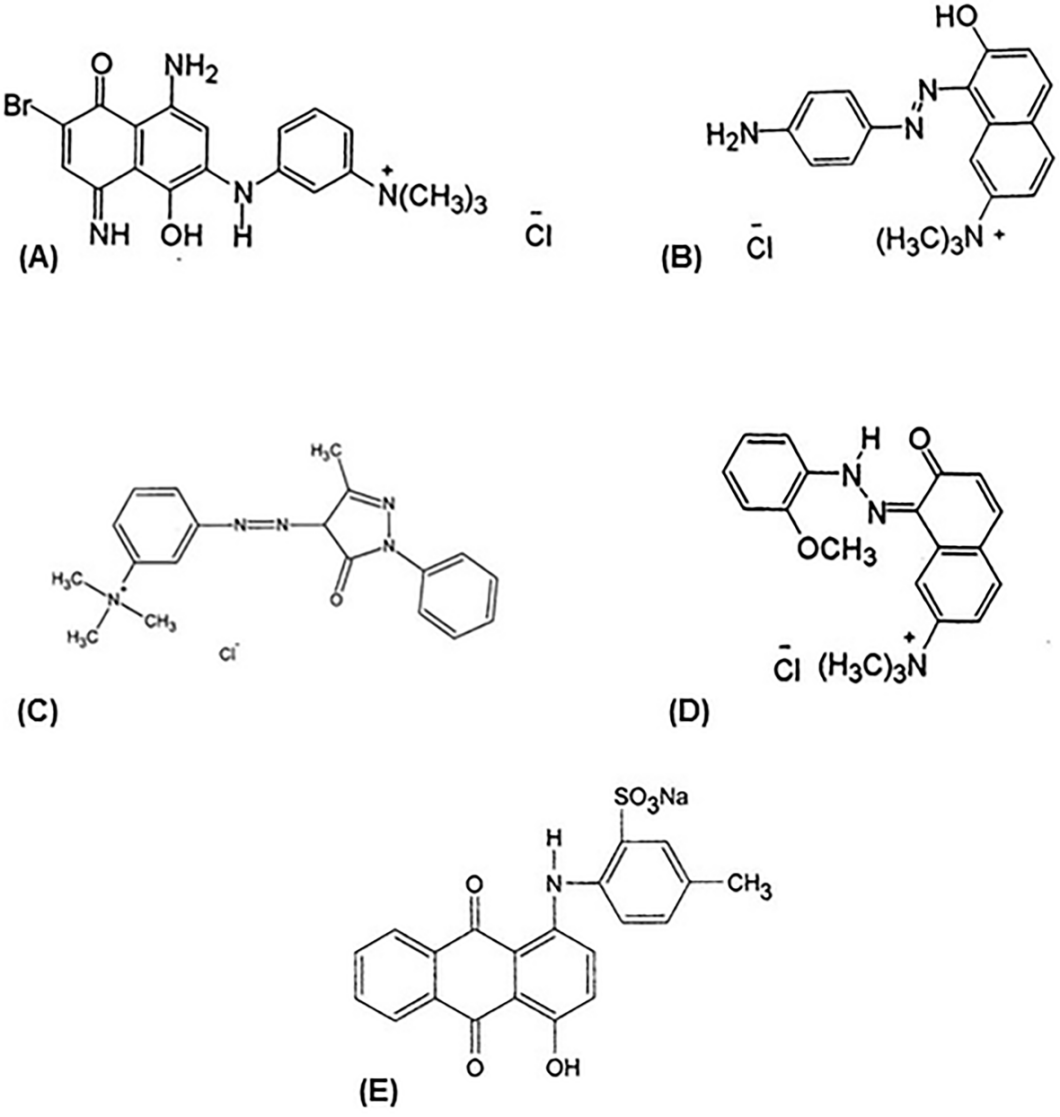
Chemical structures of the dyes present in the Arianor Ebony dye composition: **A**, Basic Blue 99 (SCCP/1437/11); **B**, Basic Brown 16 (SCCP/1165/08); **C**, Basic Yellow 57 (SCCS/1231/09); **D**, Basic Red 76 (SCCS/1385/10); and **E**, Acid Violet 43 (SCCS/1494/12).

The commercial hair dye Arianor Ebony dye (Sensient Cosmetic Technologies) at 0.32% was used for the bioassays. This dye is composed of a mixture of five dyes: Basic Blue 99 (CAS No. 68123-13-7), Basic Brown 16 (CAS No. 26381-41-9), Acid Violet 43 (CAS No. 4430-18-6), Basic Red 76 (CAS No. 68391-30-0), and Basic Yellow 57 (CAS No. 68391-31-1), and is widely used around the world (Figure 1).

### Biological materials - human cell culture

HepG2 cells (ATTCC^®^ HB-8065™) were cultivated and maintained in 25-cm^2^ culture flasks with 5 mL of Minimal Essential Medium (MEM; Gibco/Cultilab, USA) supplemented with 10% fetal bovine serum (FBS) and 0.1% antibiotic-antimycotic solution (10,000 IU/mL penicillin, 10 mg/mL streptomycin, Cultilab). Flasks were maintained at 37°C and 5% CO_2_ atmosphere until cellular confluence of ∼80%.

### Bioassays

All experiments were performed in triplicate.

#### MTT - cytotoxicity

The MTT assay (Sigma) was performed in 96-well microtiter plates, adapting the protocol established by Mosmann (16). The Arianor Ebony dye (40 mg) was dissolved in sterilized bi-distilled water (5 mL). Then, this solution was dissolved again in MEM, and the following concentrations were tested for cytotoxicity assessment: 800, 400, 200, 100, 50, 25, and 12.5 µg/mL.

For the analyses, 2.34×10^4^ cells were seeded using 100 µL of medium supplemented with FBS and incubated for 24 h. After this period, the medium was replaced by a fresh one (without FBS), in which the treatments (with concentrations between 12.5 and 800 µg/mL) were added in a final volume of 200 µL. The negative control (NC) group was composed of cells cultivated in culture media MEM (without FBS). For the positive control (PC) group, cells were also cultivated in MEM (without FBS) and then enriched with 1% Triton X-100. These control conditions were present in all experiments performed in this study. After treatment, cells were incubated for 4 h at 37°C. After this period, the culture medium was discarded and replaced by 150 µL of MTT diluted in phosphate-buffered saline (PBS) at a concentration of 5 mg/mL, and the plates were incubated for another 4 h at 37°C (15).

Then, the medium containing the MTT solution was discarded and 100 µL of dimethyl sulfoxide (DMSO) was added to each well. The plates were read in a spectrophotometer microplate reader using a 540-nm filter (Multiskan apparatus FC; ThermoScientific, USA).

The MTT assay is useful to indicate the highest concentration that assures 80% cell viability to be used in subsequent analyses (comet assay, MN test, and gene expression analyses). Moreover, inhibitory concentration inducing 50% cytotoxicity in exposed cells (IC50) was calculated.

#### Comet assay - genotoxicity

The comet assay was performed according to the protocols established by Singh et al. (17) and Tice et al. (18). For that, 5×10^5^ cells were seeded in 25-cm^2^ culture flasks and incubated for 24 h at 37°C in a 5% CO_2_ incubator. After this period, different concentrations of the dyes were added to the cultures in flasks (according to MTT results and assuring 80% viability), followed by 4 h incubation under regular cellular conditions. The NC cells were incubated with only MEM culture media and PC was performed with cells incubated with methyl methanesulfonate (MMS, CAS No. 66-27-3, Merck, USA) diluted in culture media at a final concentration of 4×10^-4^ M. After exposure, cells were washed with PBS and collected after trypsinization (with trypsin at 0.5% (Gibco)). The trypsin was inactivated with culture medium and the cells were harvested, obtaining a cell suspension. Next, 20 µL of cell suspension was mixed with 120 µL of low melting point agarose (Invitrogen Cas No. 15517-014, USA) at 0.5% and 37°C. This mixture was placed on slides previously covered with standard 1% agarose, and then the mixture was covered with coverslips. After solidification, coverslips were removed and cells were exposed to a lysis solution [10 mM Tris (Merck, Cas No. 1185-53-1), 8 g NaOH, and 10 mL of 1% (w/v) sodium lauroyl sarcosinate solution plus 1 mL of Triton X-1 00, 10 mL DMSO, and 89 mL lysis solution (pH 10) containing 2.5 M NaCl, 100 mM EDTA, 10 mM Tris, and 8 g NaOH] for at least 1 h to lyse the cells and their membranes, exposing nucleoids. All procedures were performed in the dark at 4°C. After lysis, the slides containing the nucleoids were placed in an electrophoresis chamber and covered with an alkaline buffer (pH<13) (300 mM NaOH and 1 mM EDTA solution) for 10 min to unwind DNA strands. Then, the samples were subjected to electrophoresis at 39 V, 300 mA (∼0.8 V/cm) for 20 min, neutralized in Tris buffer (0.4 M Trizma- HCl, pH 7.5) for 5 min, fixed in absolute ethanol for 10 min and stored at 4°C until analysis. Slides were stained with 50 µL GelRed^®^ solution and analyzed immediately in an epifluorescence microscope. One hundred nucleoids were scored per slide, totaling 600 nucleoids per treatment.

Nucleoids were analyzed with the CASP program (Comet Assay Software Project, RRID:SCR_007249). DNA damage was quantified by measuring the variable Olive Tail Moment (OTM), which is calculated from the product between the amount of DNA in the tail and the mean migration distance in the tail (i.e., tail length × fluorescence intensity in the tails) (19).

The damage induction factors (IFs) were calculated by comparing median values of OTM from exposed cells to median values of OTM from the corresponding NCs to evaluate the genotoxic potential of the test substance compared to NC (20).

#### Micronucleus test - genotoxicity

The MN test was performed according to the protocol established by Natarajan et al. (21) with some modifications. Approximately 5×10^5^ cells were seeded in 25 cm^2^ culture flasks and incubated for 24 h at 37°C in a CO_2_ incubator (5%). After this period, cells were exposed or not to the treatments (according to MTT results - 80% viability) for 4 h, as already described for the comet assay. NC and PC were also performed. After the treatments, the culture medium from each condition was replaced by a fresh one, and 50 µL of the cytokinesis inhibitor cytochalasin B (3 µg/mL, Merck, USA) was added to the cultures to stop cytokinesis and obtain binucleated cells. Cells were incubated for 28 h and then washed with PBS and trypsinized (with 0.5% trypsin). The trypsin was inactivated with culture medium and cells were harvested. Cell suspension was then centrifuged (5 min at 252 *g*), the supernatant was discarded, and the pellet was resuspended with hypotonic solution (1% sodium citrate). This solution was centrifuged for another 5 min at 252 *g*, the supernatant was discarded, and the pellet was resuspended with a fixation solution of formaldehyde (40%) and ethanol-acetic acid (3:1 v:v, Merck). Approximately 1 mL of cell suspension was placed on cold wet slides and air dried, and then the slides were stained with 5% Giemsa (Merck) for 8 min. Two thousand binucleated cells were scored per replica, for a total of 6000 cells per treatment. The number of micronuclei (MN), nucleoplasmic bridges, and nuclear buds was observed in an optical microscope as biomarkers of genotoxic events and chromosomal instability.

#### Gene expression analysis

For analyses, 1.5×10^5^ cells were seeded in 75-cm^2^ flasks and exposed to the dye for 4 h. Then, total RNA was extracted from cells using TRIZOL^®^ Reagent (Thermo Fisher Scientific, USA) according to the manufacturer's instructions. RNAs were quantified using the NanoVue Plus Spectrophotometer (GE Healthcare Life Science, USA), and 1 µg of each sample was reverse transcribed into cDNA using the High Capacity cDNA Reverse Transcription Kit (Thermo Fisher Scientific), following the supplier's instructions. cDNA was used as template in the polymerase chain reaction (PCR) according to Sambrook et al. (22), and the expression levels of genes associated with the apoptotic and oxidative stress pathways were investigated. Results were normalized to the reference gene β-actin. Table 1 presents the name of the genes and the oligonucleotides used for their analyses. The oligonucleotides were designed using Primer 3 Plus (https://www.bioinformatics.nl/cgi-bin/primer3plus/primer3plus.cgi). The assays were performed either in a thermocycler Mastercycler Pro S (Eppendorf, USA) or in a StepOne™ Real-Time PCR System (Thermo Fisher Scientific).

**Table 1:** Oligonucleotides sequences used in the gene expression analysis in HepG2 cells.

Gene	Primer Forward (5′-3′)	Primer Reverse (5′-3′)
*Cytochrome c*	5′-TTTGGATCCAATGGGTGATGTTGAG	5′-TTTGAATTCCTCATTAGTAGCTTTTTTGAG
*Caspase 9*	5′-CGTGGTGGTCATTCTCTCTCA	5′-TCGAAGGTCCTCAAACCTTCCTGG
*Caspase 3*	5′-GCAGCAAACCTCAGGGAAAC	5′-TGTCGGCATACTGTTTCAGCA
*Bcl-2*	5′-TTTGAGTTCGGTGGGGTCAT	5′-TGACTTCACTTGTGGCCCAG
*Fas*	5′-ACCTCTGGTTCTTACGTCTGTTGC	5′-CATGTCCTTCATCACACAATC
*Bax*	5′-TGGCAGCTGACATGTTTTCTGAC	5′-GTCCCAACCACCCTGGTCTTGG
*β-Actin*	5′-GATCATGTTTGAGACCTTCAACAC	5′-CGTCACACTTCATGATGGAGTTGACAT
*Catalase*	5′-CGTGCTGAATGAGGAACAGA	5′-AGTCAGGGTGGACCTCAGTG
*GPX-1*	5′-TTGACATCGAGCCTGACATC	5′-ACTGGGATCAACAGGACCAG
*GSS*	5′-AAGACCGAAGGCTGTTTGTG	5′-TCTTCACCCACATCCAGTGA
*GSR*	5′-ATCCATATGCAGGGACTTGG	5′- GTAGGGTGAATGGCGACTGT
*SOD-1*	5′-GATCATGTTTGAGACCTTCAACAC	5′-CTCCTTAATGTCACGCAC

### Statistical analyses

For MTT, MN assays, and comet assays, statistical analyses were performed with one-way ANOVA, followed by Tukey's comparison test (P<0.05) and non-linear regression analysis, using the software IBM SPSS^®^ v.28 (USA). For gene expression, results were statistically analyzed by one-way ANOVA, followed by Dunnet's *post hoc* test using the software GraphPad Prism v.7.05 (USA).

## Results

### MTT - cytotoxicity

Results indicated that higher concentrations of Arianor Ebony (800, 400, 200, and 100 µg/mL) induced cytotoxic effects on HepG2 cells compared with NC (Table 2). Contrastingly, lower concentrations of the analyzed substance (50, 25, and 12.5 µg/mL) did not induce statistically significant cytotoxic effects to exposed cells compared with NC.

**Table 2:** Cell viability based on the colorimetric MTT assay performed with HepG2 exposed to different concentrations of the Arianor Ebony dye (treatments).

Treatments	Viability (%)
Negative control	100±0.067
Positive control	12.4±0.005*
800 μg/mL	12.4±0.009*
400 μg/mL	12.2±0.047*
200 µg/mL	27.7± 0.026*
100 μg/mL	29.7±0.039*
50 μg/mL	97.6±0.096
25 μg/mL	98.7±0.096
12.5 μg/mL	98.2±0.056

Data are reported as mean and standard deviation. Experiments were performed in triplicate. *P<0.05 compared to the negative control (ANOVA/Tukey's test). Positive control: 1% Triton X-100.

The IC50 value for Arianor Ebony dye was found to be 81 µg/mL for a 4-h exposure. Based on the preliminary results, the three lowest concentrations were used to perform the comet assay, MN test, and gene expression analyses.

### Comet assay - genotoxicity

**Figure 2:**
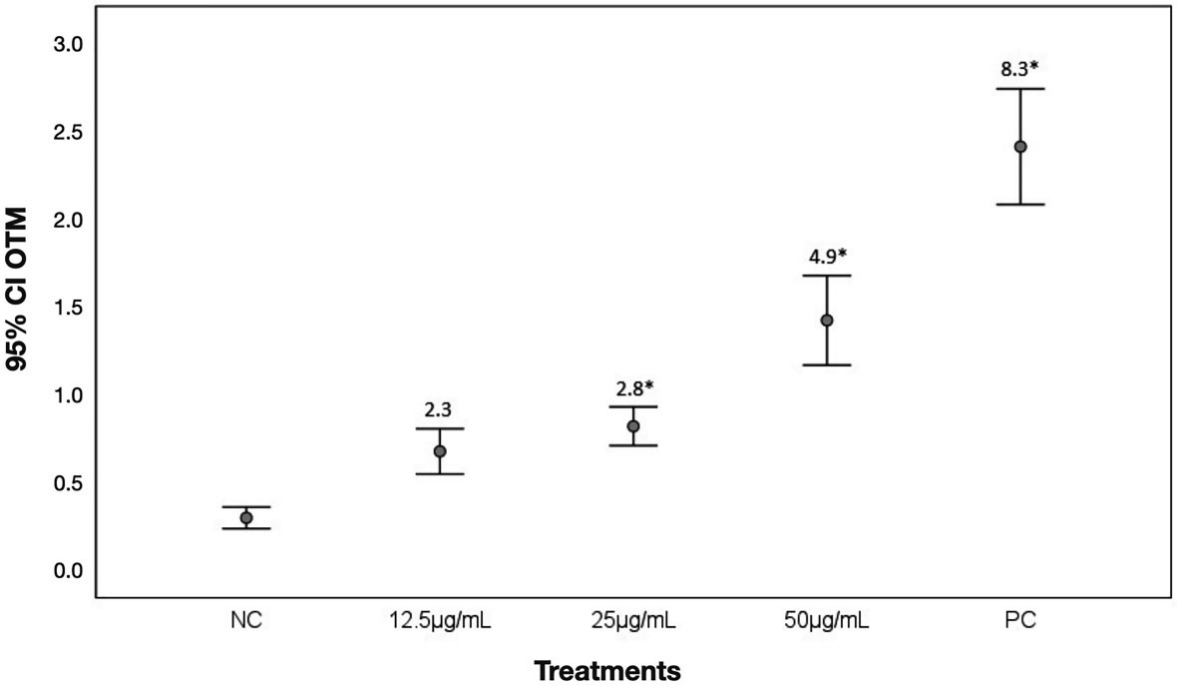
Genotoxic effects in HepG2 cells assessed with the comet assay. Data are reported as the mean OTM (Olive Tail Moment) and 95% confidence intervals of 600 scored nucleoids per Arianor Ebony dye concentrations. NC: negative control; PC: positive control. The induction factors (IF) shown in the graph are the quotient of the median values of OTM of exposed cells and PC cells and the median values of OTM of the NC cells. *P<0.05 compared to NC according to Tukey's test.

A dose-response curve of cells exposed to Arianor Ebony could be observed, with statistically significant effects of doses from 25 to 50 µg/mL. IF of exposed cells were also two- to five-fold higher than NC values (Figure 2), indicating genotoxic effects induced by the dye.

### Micronucleus test - genotoxicity

All tested concentrations of Arianor Ebony dye induced significantly more MN in HepG2 cells compared to NC, and recorded values were comparable to those obtained in PC cells, indicating genotoxicity induced by the dye (Table 3). The other parameters (nuclear buds, nucleoplasmic bridges) analyzed were not statistically significant compared to NC, except for the 25 µg/mL concentration, which induced significantly more nuclear buds.

**Table 3:** Cytokinesis-block micronucleus test performed with HepG2 cells exposed to different concentrations of Arianor Ebony dye (treatments).

Treatments	MN	NBUDs	NPBs
Negative control	31.6±2.51	6.0±2.64	1.33±0.57
Positive control	87.3±4.04*	12.0±2.00	10.67±3.21*
50 μg/mL	83.0±9.53*	8.0±3.00	7.67±3.05
25 μg/mL	80.0±4.58*	17.0±6.10*	7.67±4.04
12.5 μg/mL	81.0±3.21*	11.33±5.03	4.33±1.52

Data are reported as mean and standard deviation. Experiments were performed in triplicate. *P<0.05 compared to the negative control (NC) (ANOVA followed by Tukey's test). MN: micronucleus; NBUDs: nuclear buds; NPBs: nucleoplasmic bridges; Positive control: 1% Triton X-100.

### Gene expression analyses

**Figure 3:**
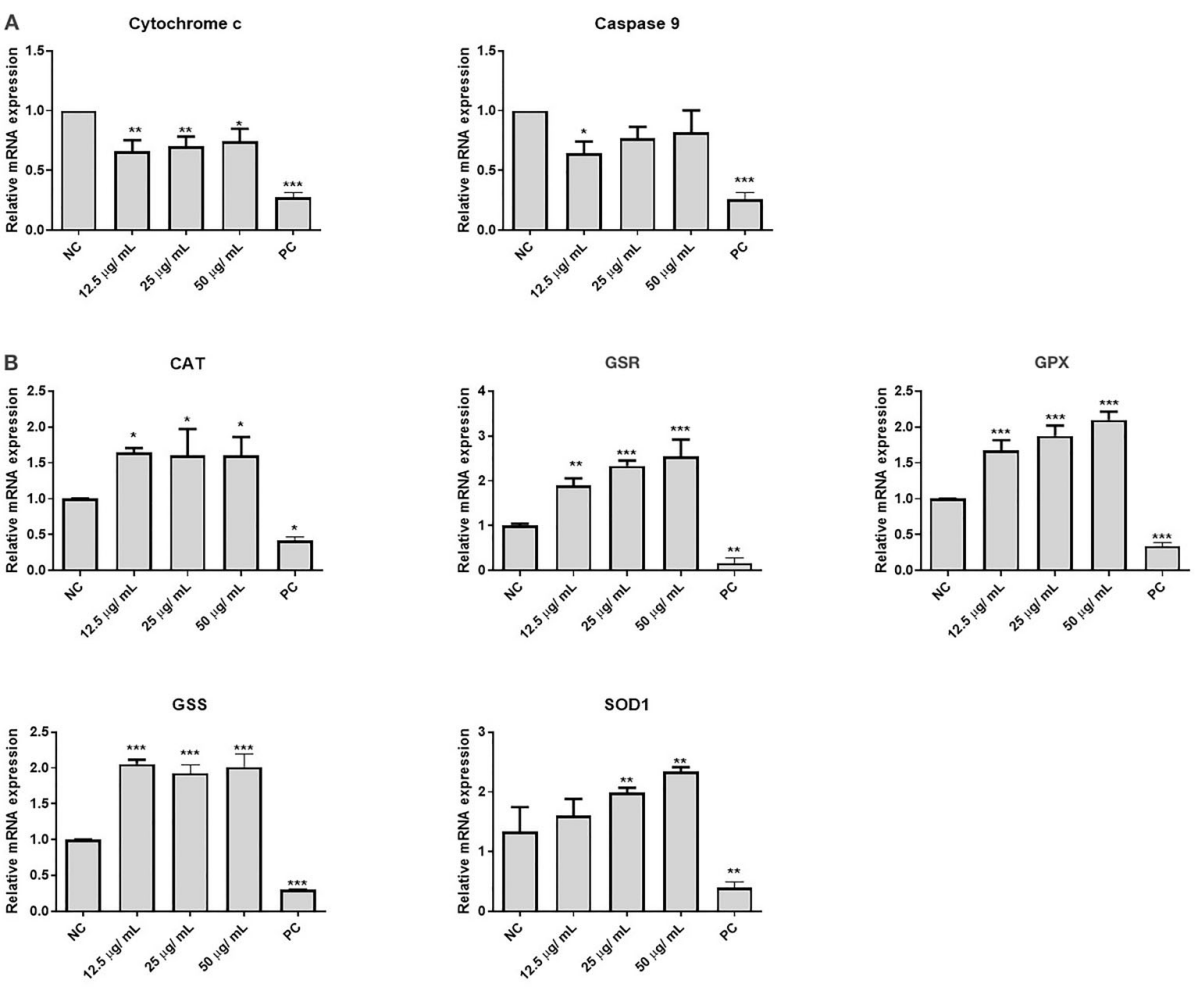
Relative transcription levels of the pro-apoptotic genes *cytochrome c* and *caspase 9* (**A**) and antioxidant molecules catalase (CAT), glutathione-disulfide reductase (GSR), glutathione peroxidase 1 (GPX), glutathione synthetase (GSS), and superoxide dismutase 1 (SOD1) (**B**). The analyses were performed using HepG2 cells exposed to the Arianor Ebony dye. Data are reported as means±SE from at least three independent experiments. *P<0.05, **P<0.01, ***P<0.001 compared to NC (one-way analysis of variance (ANOVA) followed by Dunnett's test). NC: negative control; PC: positive control.

A reduction in transcript levels of *cytochrome c* and *caspase 9* was found compared to the NC expression level (Figure 3A). For cytochrome c, the presence of the dye decreased the gene expression by 27, 21, and 16% at concentrations of 12.5, 25, and 50 μg/mL, respectively. For *caspase 9,* there was a reduction of 44, 33, and 38%, respectively, for the same concentrations. For the other investigated apoptotic genes (Table 1), the results were not statistically significant compared to NC and are not presented. Moreover, gene expression was significantly increased for catalase (*CAT*), superoxide dismutase 1 (*SOD1*), glutathione synthetase (*GSS*), glutathione-disulfide reductase (*GSR*), and glutathione peroxidase 1 (*GPX1*) in all investigated groups of cells exposed to different concentrations of the Arianor Ebony dye compared to NC (Figure 3B). The average increase in gene expression of the antioxidant genes was more than 100% for all investigated conditions. PC cells, exposed to detergent, presented decreased expression of all the investigated genes.

## Discussion

Hair coloring products may contain several substances with genotoxic, mutagenic, and/or carcinogenic potentials (23). Many of these substances contain aniline metabolites that can induce chromosomal aberrations, which correlate with changes in gene expression that may cause cancer and other diseases (24). Due to the increasing popularity of hair dyes and their economic importance, it is crucial to assess the toxic potential of these products because they have become a public health concern (25). Studies support a strong correlation between the use of some hair products and many types of diseases, such as breast, ovarian, bladder, lung, and hematopoietic cancers, lymphoid neoplasms, soft tissue sarcoma, and childhood tumors (26). Arianor Ebony dye, investigated in this study, is composed of a mixture of azo and anthraquinone dyes that may induce different mechanisms of toxic action. Results obtained with the MTT assay showed that Arianor Ebony induced cytotoxic effects in HepG2 cells from the 50 µg/mL concentration. This could be related to the ability of the aromatic structure to produce free radicals, which disrupt mitochondrial function (27).

In *in vivo* systems, azo dyes may be metabolized into more toxic compounds via direct oxidation of the azo structure, resulting in highly reactive electrophilic diazonium salts (26). These chemicals are also metabolized by the human gut microbiota, which reduce and/or cleave azo structures by the azoreductase activity present in several bacteria. This sometimes contributes to the formation of even more toxic compounds and may collaborate with the production of aromatic amines (28). A problem of anthraquinones is their capacity to disrupt the mitochondria electron transport chain, which modulates the oxidative mechanisms and could produce a semi-quinone radical and reactive oxygen species (ROS) (27), which damages cellular metabolism and homeostasis. These free radicals can oxidize the cellular membrane and macromolecules, resulting in the loss of cellular structural integrity, ultimately leading to cell death (29). In many hair dyes, free radicals act as couplers and react with precursors, such as para-phenylenediamine (PPD), inducing apoptosis and generating ROS in human cells through the association of *caspase 3/7* and the initiators *caspase 8* and *9* (30).

In both the comet assay and the MN test, the Arianor Ebony dye at concentrations below 50 μg/mL induced genotoxic effects in HepG2 cells. Other studies performed with hair dyes that are also present in the black dye have indicated genotoxic damage in human cells (14). The comet assay has been used as a sensitive biomarker to detect DNA strand breaks, alkali-labile sites, and cross-linking between DNA-DNA or DNA-protein. Tsuboy et al. (31) suggested that azo dye radicals can be metabolized by cytochrome P450 enzymes, generating aromatic amines, which can be converted to *N*-hydroxylamines, then to *O*-acetylated or sulfated ions, and finally to reactive nitrenium ions, which can generate DNA damage and induce carcinogenic effects in some target organs. Kilinc and Kilinc (32) demonstrated that azo dye metabolites also generate NO_2_ and -OH radicals, which can damage DNA and other cellular components. Azo dyes can be oxidizing agents because they can be reduced to hydrazines and primary amines (33).

The MN test found a significantly higher percentage of MN in HepG2 cells exposed to Arianor Ebony dye compared to NC. According to Wollin and Gorlitz (34), the interaction between the genetic material and azo and anthraquinone dyes can be related to electrophilic structures, which are able to form covalent bonds with DNA. An explanation is that azo dyes can be activated by peroxidase and the resulting metabolites can form guanosine DNA adducts, which are genotoxic *in vitro* (33). According to Chequer et al. (35), the toxicity of azo dyes is related to high doses that can be lethal, causing formation of micronuclei, DNA fragmentation, and apoptosis in human hepatoma cells. Other authors also reinforced the toxic effects of hair dyes through the analyses of DNA interactions (36), which could be considered a serious concern in the modern world. This has been evidenced by MN analysis in hairdressers; occupational exposure to the dyes considerably increases the average number of MN in those professionals in a time-dependent manner (37). This could partly explain the increased cancer risk of hairdressers, which is proportional to their exposure time (24).

In our genotoxic investigation of Arianor Ebony dye, the comet assay and MN test revealed significant effects in all tested concentrations. Brambilla et al. (38), investigating the genotoxic potential of an azo dye in the same cell line, also found positive results in both comet and MN tests. These authors concluded that the DNA strand breaks leading to comet formation were only partly repaired and induced persisting chromosomal alterations detectable as MN, which could be an explanation for the results obtained in the present research.

To verify the correlation between the cellular effects of the Arianor Ebony dye and molecular dysfunctions, we investigated the gene expression of relevant molecules in the apoptotic and oxidative molecular pathways. In our cellular model, most genes related to apoptosis did not present relevant changes in exposed cells compared to NC, which may be due to the protein half-life produced by each investigated gene controlling the mRNA level. Proteins with longer half-lives do not need extra rounds of translation as observed in the assays. Exceptions were observed for the *cytochrome c* and *caspase 9* genes, whose expressions were down-regulated in cells exposed to the dyes compared to NC. The significant decrease of *cytochrome c* and *caspase 9* gene expressions suggested the presence of a mechanism contributing to HepG2 cell survival, avoiding the assembly of the apoptosome and reducing death rates as observed in the MTT test results. Apoptosome assembly is strictly connected to the availability of the cytochrome c and caspase 9 proteins (39). Our gene expression analyses of antioxidant enzymes also suggested the presence of metabolic routes that lead to ROS production based on the increased transcription levels of protective and antioxidant enzymes (catalase, GPX, GSS, GSR, SOD1), which combined with the toxic cellular environment induced by the hair dye favors homeostasis disruption and the combined deleterious effects observed in the present study. Moreover, our results corroborated those of other studies that demonstrated that some specific components of hair dyes can modulate *cytochrome c* and *caspase 9* genes due to increased ROS production (5,30).

Overall, our results indicated acute and specific toxic effects of Arianor Ebony dye to the human cell line HepG2, where even the lowest tested concentration induced genetic damage, which may be related to its azo and anthraquinone structures.

Cell lines have been successfully used as alternative test systems in *in vitro* bioassays to assess toxicological molecular and cellular effects of pure substances or mixtures. Those effects may culminate in damage to organisms, populations, and even ecosystems, and are the first alert of the risks to human and environmental health (40). However, the results obtained in *in vitro* investigations are not enough to determine the effects in systems of higher levels of organization. Thus, additional studies should be performed with other models to better understand the toxic pathways of substances such as Arianor Ebony. Moreover, a combination of *in vitro* results with some occupational investigations of the exposed population could increase the understanding about the real hazard (and possible risk) of this dye.

In the specific case of hair dye formulations, such as the black color (including Arianor Ebony), it is also necessary to consider the synergistic effects of the multiple agents in their composition, their degradation, and the possible effects to the environment. The Arianor Ebony dye may be hazardous to human health. However, further investigations are needed to truly understand the mechanisms of action of this dye, considering its toxic potential to the exposed population and even to the environment.
